# *NEISSERIA SUBFLAVA* PERITONITIS: CASE REPORT

**DOI:** 10.1590/0102-6720201700020018

**Published:** 2017

**Authors:** Changhua CHEN, Ping-Fang CHIU, Jen-Shiou LIN

**Affiliations:** 1 Changhua Christian Hospital, Department of Internal Medicine, Changhua, Taiwan

**Keywords:** Peritonitis, Treatment failure, Catheter-related infections

## INTRODUCTION

Peritonitis is a common and serious complication of continuous ambulatory peritoneal dialysis (CAPD)^1^, and peritonitis is still the major leading cause of death in around 16% of patients receiving it in continuous ambulatory peritoneal dialysis^1^. Its usual etiological agent in peritonitis is a gram-positive coccus, while *Neisseria subflava* rarely causes peritonitis.

## CASE REPORT

A 74-year-old man who had had uremia since 2010 and was receiving maintenance CAPD was admitted because of fever, nausea, vomiting, diffuse abdominal pain, and opaque dialysis effluent one day prior to admission. On admission, no rebound tenderness was observed. His serum white blood cell count was 7.000/µl and serum creatinine level was 14.49 mg/dl. Abdominal plain radiography showed no evidence of ileus or bowel obstruction. *N. subflava* from dialysate was identified on matrix-assisted laser desorption ionization-time of flight mass spectrometry (bioMerieux, Hazlewood, Mo.). A susceptibility test for *N. subflava* was performed by using the bioMérieux VITEK 2 system (bioMerieux, VITEK 2 system, Hazlewood, Mo.). The minimum inhibitory concentration of ceftriaxone was 0.094 µg/ml by Etest (AB Biodisk, Sweden). His blood cultures returned negative results for any pathogens. The patient experienced a brief improvement in symptoms, followed by symptom recurrence. Subsequent evaluation of the effluent showed a persistent elevation of the white blood cell count from 6624/µl to 1890/µl in three days. His condition was compatible with refractory peritonitis according to the recommendations by the International Society Peritoneal Dialysis Peritonitis (ISPD)^1^. The peritoneal dialysis catheter was removed on admission day 7. Thereafter, he received hemodialysis three times per week. Intravenous ceftiaxone at 2000 mg daily was prescribed for 21 days. He was discharged on admission day 22 with a stable clinical condition.

## DISCUSSION

We disclose three important issues on CAPD-associated *N. subflava* peritonitis. First is the timing for the removal of the peritoneal dialysis catheter and reinsertion of a new catheter. The ISPD recommends at least five days after appropriate antibiotics and failure of cleaning the CAPD effluent (1C)^1^, and a minimum interval of 2-3 weeks between catheter removal and reinsertion of a new catheter^1^. The second is the administration duration of effective antibiotics. The ISPD recommends that non-*Pseudomonas* gram-negative peritonitis be treated with effective antibiotics for at least three weeks (2C)^1^, in contrast to two weeks as recommended by the Infectious Diseases Society of America^2^. The third is the source of infection. The present case might be due to touch contamination, exit-site infection, or possibly a dental source from periodontal disease or an oropharyngeal source^3^ ^,^ ^4^. Considering the occurrence of CAPD-associated peritonitis, the patient’s aseptic technique and home circumstances should be reviewed. In addition, potential problems were identified in the patient’s hand-washing technique and lack of face mask use. Although *N. subflava* peritonitis is rarely reported, we emphasize that compliance for the aseptic technique and standard procedure is critical in CAPD patient care.
